# Supplementary material for: How social networks affect the repression-dissent puzzle
Source: PLoS One. 2021 May 6;16(5):e0250784. doi: 10.1371/journal.pone.0250784 (PMC8101725; doi:10.1371/journal.pone.0250784)
Supplement: S1 File — (PDF) [file pone.0250784.s001.pdf]

S1 Repression-Dissent Puzzle

Many studies find that repression decreases protest. For example, repression of protests in Iran in 1978-1979 decreased protests one week later [6]. Panel data from Peru and Sri Lanka from 1955 to 1991 finds that government repression decreases dissent [2]. The effect does not appear to be particular to one country, according to a study of twenty-four non-democracies [3]. A panel study of 56 countries, democracies and non-democracies, from 1958 through 1977 also finds a negative effect of repression [35]. Repression can decrease protest when associated with other contextual features, such as elite unity or the phase of the protest cycle [40].

Many studies find that repression increases protest. For example, repression of protests in Iran in 1978-1979 increases protests six weeks later [6]. The effect does not appear to be particular to one country: across twenty-four non-democracies, increased repression at the beginning of a protest cycle leads to more mobilization [3].[1] Repression also facilitated mobilization in the Philippines (1986) and then Burma (1988) [40]. In six Latin American and three African countries, repression in the 1970s through 1990s increased protest [41]. Repression in West Germany, Czechoslovakia, and Palestine increased protest [37]. Even perhaps the most extreme repression, indiscriminate massacres of protesters, seems to increase the size of subsequent mobilizations [28].

Many studies find that repression has no effect on protest. Repression of ethnopolitical groups does not affect the probability they mobilize [9], nor did it affect mobilization in Northern Ireland or West Germany [38]. A historical study of collective violence in France similarly finds no correlation [11]. There may also be no correlation because any protest that does occur is the result of the failure of state preventative repression [7].

These inconsistent findings have not become more consistent with more data. [13] relies on the Integrated Data for Events Analysis dataset, which records 10 million events from more than 200 countries from 1990 through 2004 [14]. [7] merges global rainfall data with data on social conflicts in Africa and protests in the United States over a 30 year period to argue that preventative repression decreases protest [15, 16]. [83] finds that correlates of repression are sensitive to model form and the dataset used. Daily data on protest and repression during the Arab Spring suggests that repression increased the number of protests on subsequent days [12].

S2 Social Networks and the Decision to Mobilize

S2.1 Participation Thresholds

An individual's decision to protest or not is often modeled as a weighting of the costs and benefits of participation or not participating [2]. Costs and benefits are approximated by observing how many people protest, and individuals decide to protest if this number surpasses an internal threshold [13].

One advantage of thresholds is that they reduce a complicated decision function to one dimension. Variables that affect bystanders, such as the probability of arrest [21] or ideological distance from current protestors [51], certainly contribute to the number of people protesting. Whether or not a bystander becomes a protester despite a protest being dangerous or ideologically distant is going to depend on a multitude of other variables, such as risk tolerance or emotional responses [23], that the researcher will have a difficult time specifying. Assigning individuals a participation threshold captures these heterogenous dynamics without having to model them: if a protestor does not

[1] That paper finds a negative relationship holds only "if the authorities are able simultaneously to prevent challengers' media access and to inhibit the formation of politically-oriented social networks."

care so much that a protest is dangerous, full of ideological dissimilars, or will not change policy, he or she has a low threshold, and vice-verse if those variables do make the bystander cautious.

That protest is a complex contagion also favors a threshold approach [11]. A simple contagion phenomena may spread based only on contact between two individuals, whereas complex contagion requires that an individual receive exposures from multiple sources. A disease is a simple contagion, for example, because one person can infect another; outcomes resulting from a combination of interdependent decision making and network effects, such as protest or technology adoption, are complex contagions. In a simple contagion, activated individuals with very many connections make a behavior much more likely to spread [25]; in a complex contagion, local contacts matter much more.

S2.2 Estimating Others' Participation

Calculating costs and benefits requires information about the outcomes, and that information can come from various sources. A prime source of this information is an individual's social network, the interpersonal connections he or she maintains with other people. That is, media reports of a protest size are assumed to carry no weight: a news report saying 100,000 people protested is meaningless compared to knowing a large percentage of one's connections protested. This assumption is perhaps extreme in a democracy but captures the media environment of many countries, especially those with revolutionary protests. If the decision to protest is based on whether or not a certain threshold of one's connections have protested and the only information about that number comes from observing one's network, then understanding how information about protest participation diffuses through a network is equivalent to understanding how protests attain a certain size.

The empirical literature supports the assumption that connections influence individuals' protest participation. In the United States Civil Rights Movement, knowing a friend who joined (left) a Freedom Summer organization strongly influenced an individual's decision to join (leave) that organization [14]. In the German Democratic Republic in 1989, personal networks explained protest participation more than membership in organizations or selective incentives [15]. Talking to others who participate in protests also made Egyptians in 2011 more likely to protest [28].

S2.3 Three Network Types

Three characteristics define social networks: more local clustering than chance allows (many mutual friends), short average path links ("small-world"), and a very skewed degree distribution (a few very popular people) [29]. A model of protest diffusion should therefore operate on a network that encapsulates these three features.

Unfortunately, as Table A1 shows, there is not a model that combines all three. The Watts-Strogatz model creates highly clustered networks with long average path lengths (a ring or lattice network), highly clustered and short networks (the small-world network), or lowly clustered and short networks (random networks) as a function of the probability of rewiring edges between nodes [19]. But because it starts with a network in which all nodes have the same degree, it never reproduces the skew of influence that characterize real-world social networks. The Barabási-Albert model, associated most closely with [20], uses the preferential attachment mechanism of [32] to generate networks that replicate the short paths and skewed degree of social networks. That model does not, however, generate clustering in line with social networks, though its clustering is greater than random networks. The Holme-Kim model modifies the Barabási-Albert one by introducing the possibility that a new node's edge can connect

to another node with which it has a mutual friend. This modification can be tuned to vary the amount of clustering in a network, and it preserves the short paths and skewed degree of a Barabási-Albert network [21].

Table A1. No Model Combines all Features

Feature	Watts-Strogatz	Barabási-Albert	Holme-Kim
Local clustering	✓	No	✓
Short paths	✓	✓	✓
Skewed degree	No	✓-	✓-

While it would appear that the Holme-Kim model is preferable, it does not generate degree skew as extreme as observed in social network data. With both the Barabási-Albert and Holme-Kim models, the skew will have a scaling exponent of approximately 3, whereas human social networks have a skew of approximately 2.3 [64]. Varying the degree skew in those models would require modifying the preferential attachment mechanism, a task that would be its own project and is therefore outside the scope of this one.

Instead, we generate a sequence of numbers from Barabási-Albert distributions whose exponent we vary; varying this exponent allows us to control the skew of degree centrality. For the rest of this paper, "Barabási-Albert" therefore means a network generated not via preferential attachment but by randomly assigning nodes a number of edges drawn from a Barabási-Albert distribution whose skew we control.[2]

S3 Mass Mobilization Dataset Robustness

Figures A1 and A2 show that the repression-dissent puzzle is not sensitive to the model specified in the manuscript for the Mass Mobilization dataset. For Figure A1, we use the same sampling procedure and model as before except add variables for protester demands. The dataset indicates whether a protest is over labor, land, police brutality, political demands, prices, leadership removal, or social issues.

We considered adding a variable for protester identity, but the strings describing those are highly collinear with the protester demands. For example, protester identity usually involves words like "employee" or "worker" when demands are classifier as about labor. We therefore did not run a model including protester identity.

For Figure A2, we use the same independent variables as in Figure 1 but make the dependent variable the duration in days of protests. The repression-dissent puzzle persists.

We did not perform the same analysis for the Mass Mobilization in Autocracies Dataset because it does not include event-level data on protester demands or identity.

[2]For detail on Barabási-Albert distributions, see [35] or [36]

Fig A1. Controlling for Protester Demands

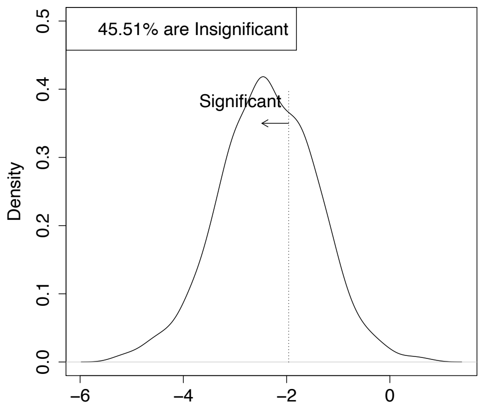

(a) Correlation with Arrest

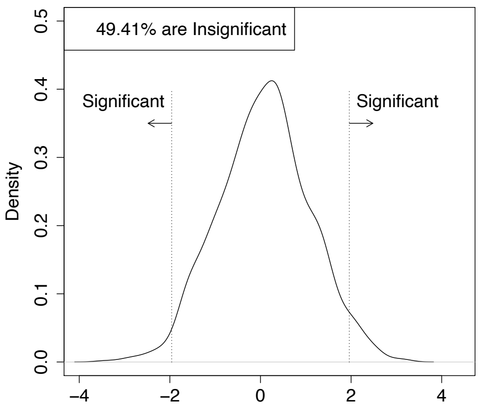

(b) Correlation with Killing

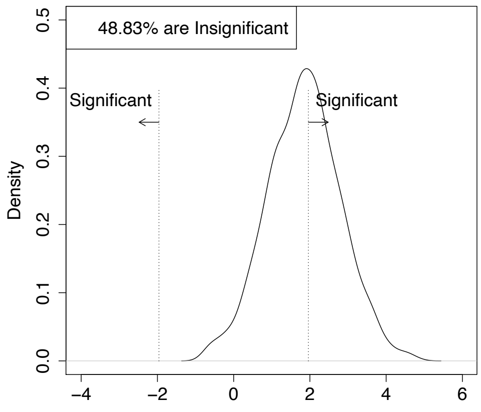

(c) Correlation with Shooting

Note: Controlling for protester demands in the Mass Mobilization dataset does not affect the repression-dissent puzzle.

Fig A2. Repression's Effect on Protest Duration

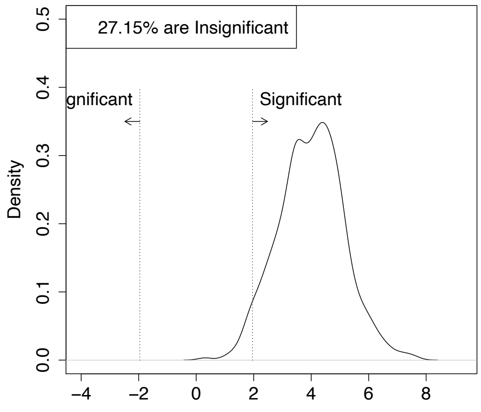

(a) Correlation with Arrest

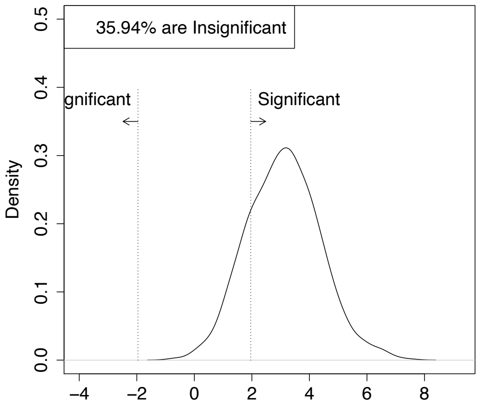

(b) Correlation with Killing

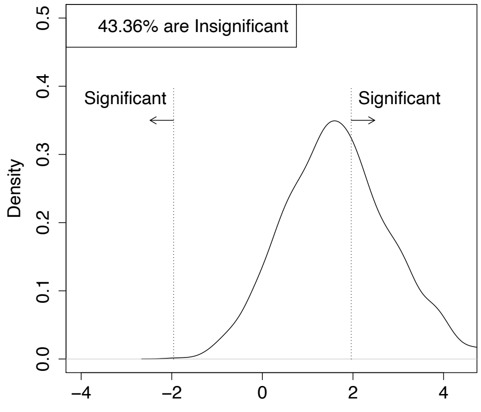

(c) Correlation with Shooting

Note: The repression-dissent puzzle also exists when considering a protest's duration as the outcome.

S4 Robustness Checks

One concern is that protests are initially large, so contradictory findings are driven by the variation in initial protest size. Figure A3 restricts analysis to only trials where the final and initial protest size differ.

Figure A4 keeps only those trials for which the initial protest size is less than or equal to the median for each network type and the protest grows. (Including protests that do not grow finds that repression has no statistically significant effect approximately 90% of the time across each network type.) The repression-dissent puzzle still emerges, though not for Holme-Kim networks in the latter subset.

Fig A3. Results Hold When Restricting to Protests Whose Size Changes

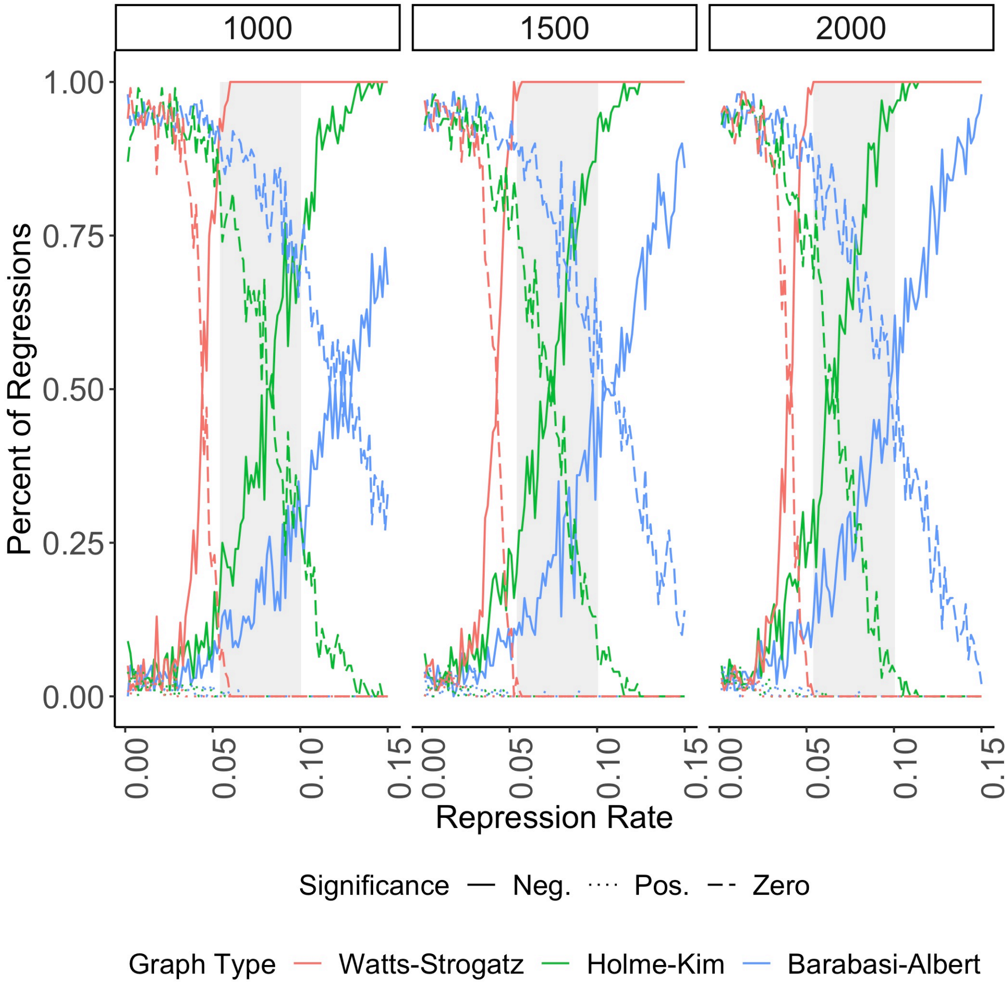

Note: The y-axis is the percent of regressions which find no relationship or a statistically significant, positive or negative, one. The facet label is the size of the sample.

Figure A5 replicates Figure 3, using a negative binomial estimator of untransformed final protest size. We also used a negative binomial model for subsets of data where network size changed and at the most realistic values of the parameter space; results match and so are not shown here. The repression-dissent puzzle is not an artifact of using ordinary least squares regression.

To confirm that our model does not recreate the puzzle by generating contradictory

Fig A4. Results Hold When Restricting to Protests Less Than or Equal to Median Size and Size Changes

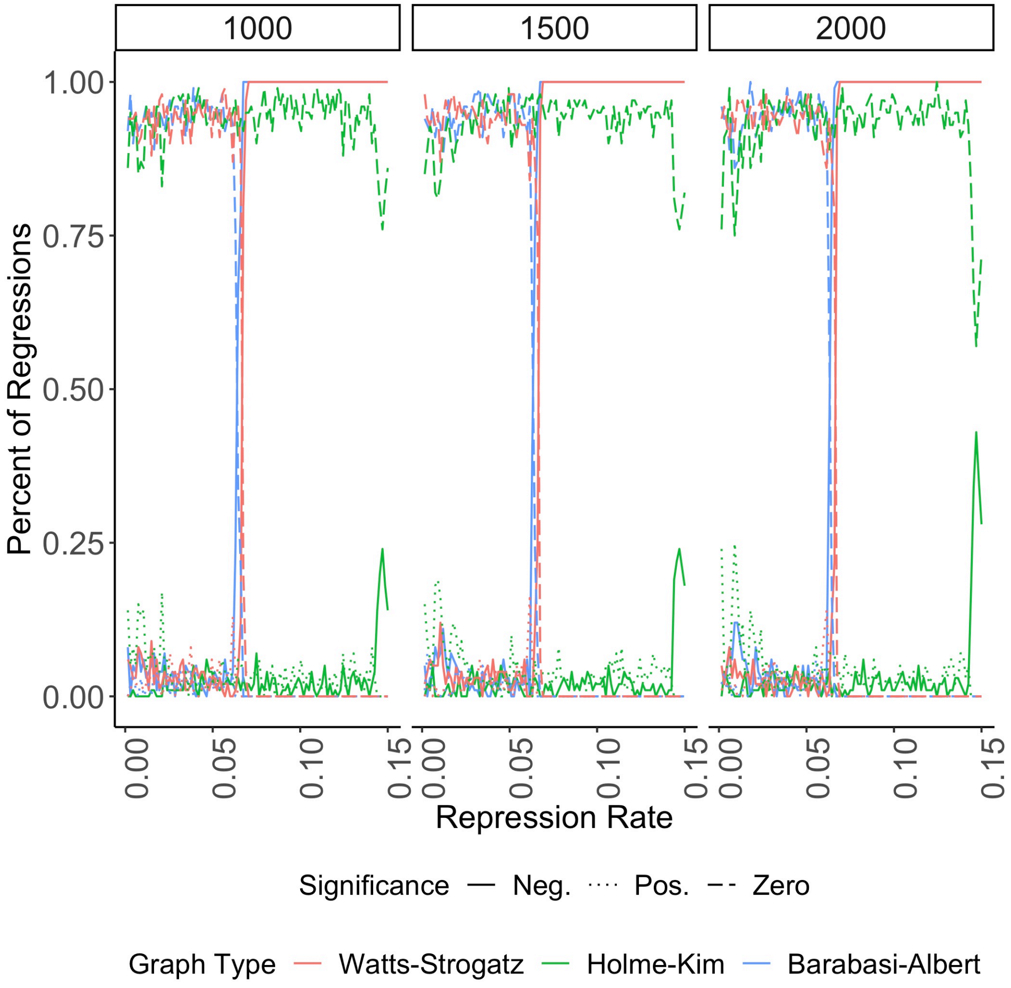

Note: The y-axis is the percent of regressions which find no relationship or a statistically significant, positive or negative, one. The facet label is the size of the sample.

results for all variables, we analyze the non-repression variables from Equation 1 and the two threshold variables later used. Figures A6-A8 show these results for regressions with 1,000 observations, using the original repression specification, and all values of r. Results are grouped by variables at the node, neighborhood, and global level. Across all network types, there are consistent results for the average threshold of initial protesters ($-$) and protest's initial size (+). Barabási-Albert and Holme-Kim networks have the same relationship for initial neighborhood clustering (+) and global density ($-$). All three networks find contradictory results for the degree of initial protesters, clustering within initial protesters, the average threshold of neighbors of initial protesters, and global clustering.

Fig A5. Results Hold With Negative Binomial Estimation

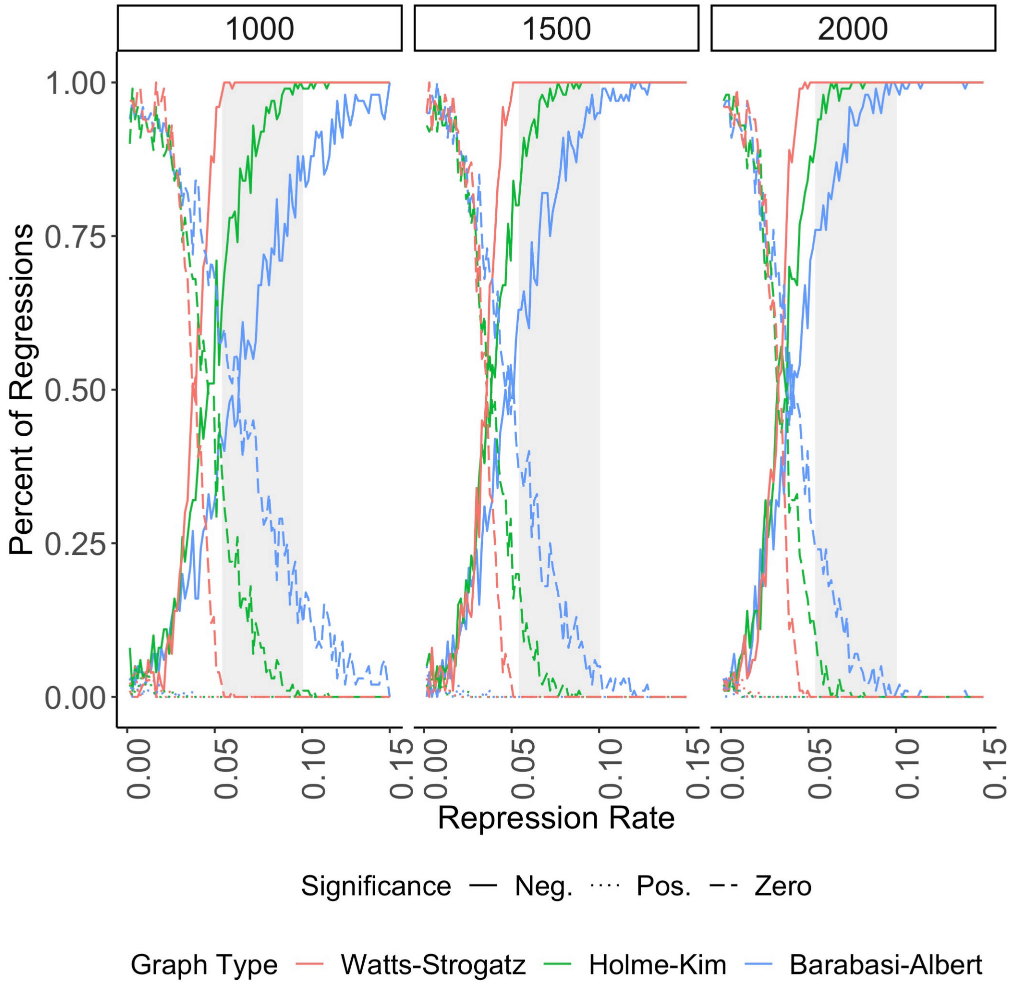

Note: The y-axis is the percent of regressions which find no relationship or a statistically significant, positive or negative, one. The facet label is the size of the sample.

Fig A6. Results for Node Variables

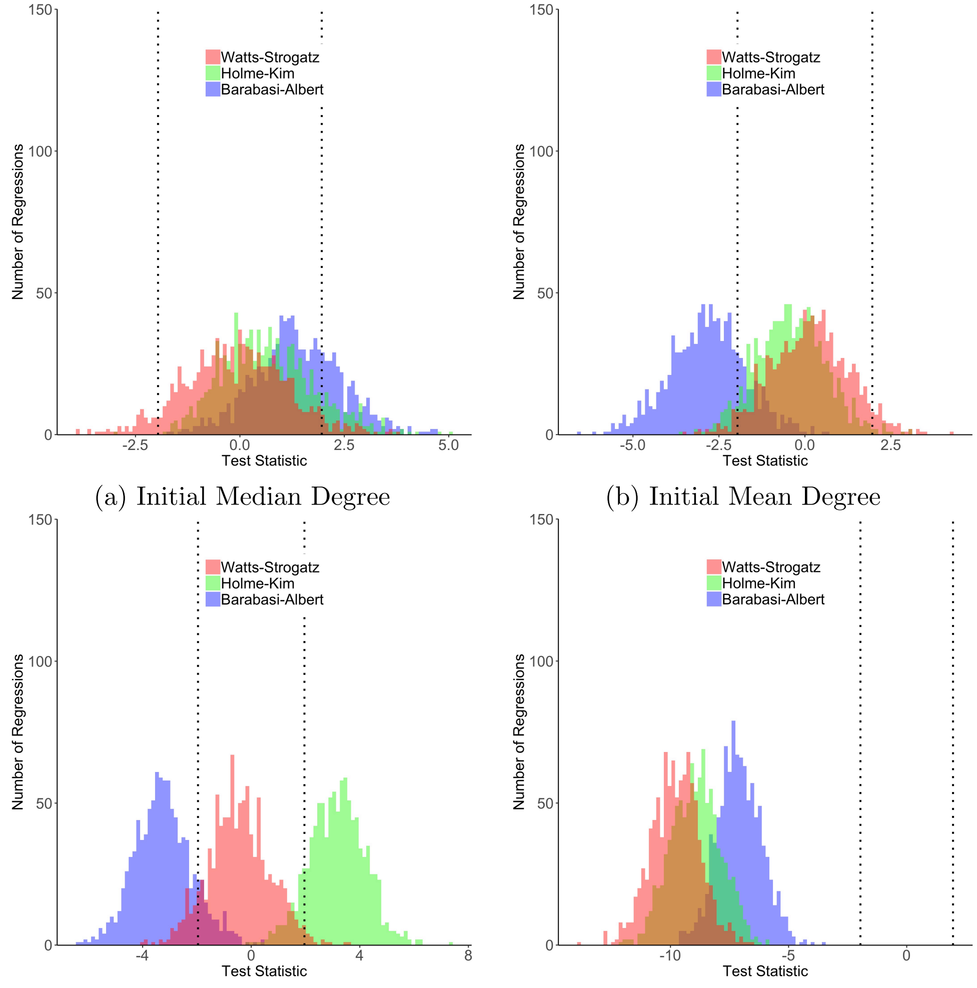

(a) Initial Median Degree
(b) Initial Mean Degree
(c) Initial Protester Clustering
(d) Initial Mean Threshold

Note: Results for other variables from Equation 1 and threshold variables. 1,000 samples, all values of r. (a)-(b) Inconsistent results for the out-degree of initial protesters. (c) Clustering within initial protesters has different effects depending on the network type. (d) Regardless of network type, increased participation thresholds for initial protesters correlates with smaller protests.

Fig A7. Results for Neighborhood Variables

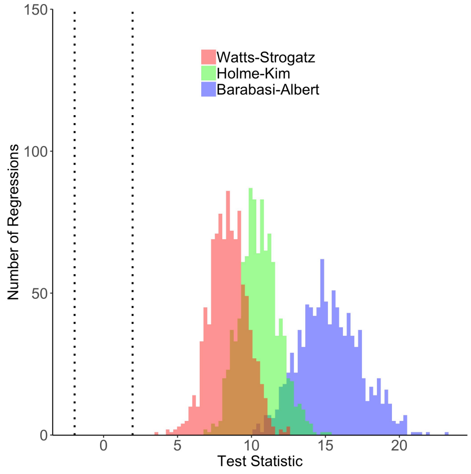

(a) Initial Size

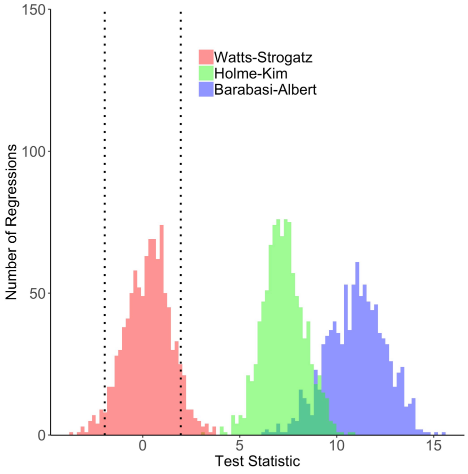

(b) Initial Neighborhood Clustering

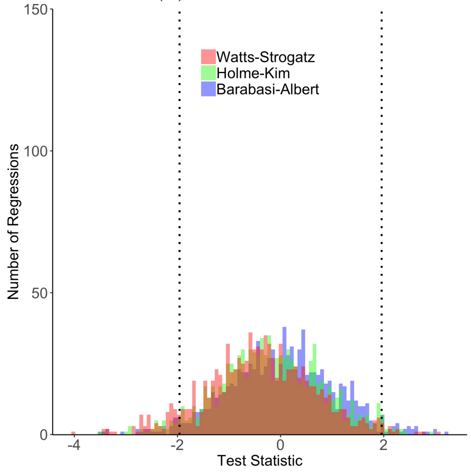

(c) Initial Neighborhood Threshold

Note: Results for other variables from Equation 1 and threshold variables. 1,000 samples, all values of r. (a) Larger initial protests strongly correlate with larger final protests. (b) Greater clustering of initial protesters' neighbors has consistent effects for Holme-Kim and Barabási-Albert networks, not for Watts-Strogatz. (c) Inconsistent results in all networks for thresholds of neighbors.

Fig A8. Results for Global Variables

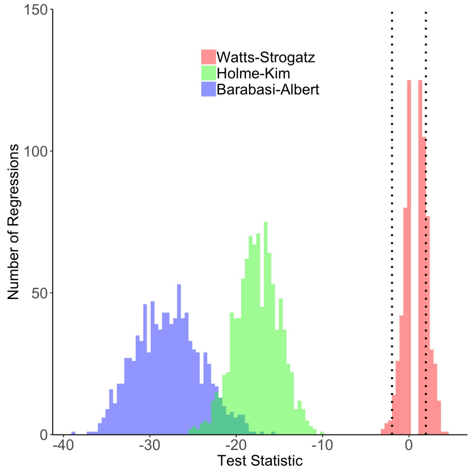

(a) Global Density

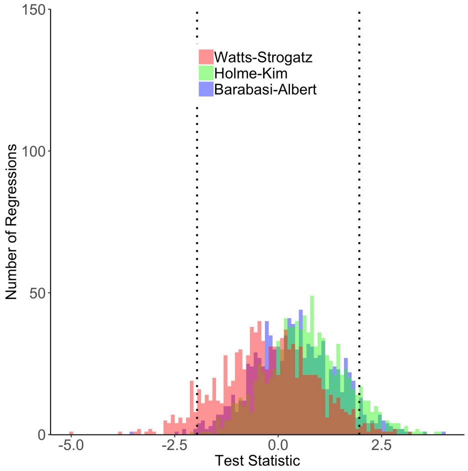

(b) Global Clustering

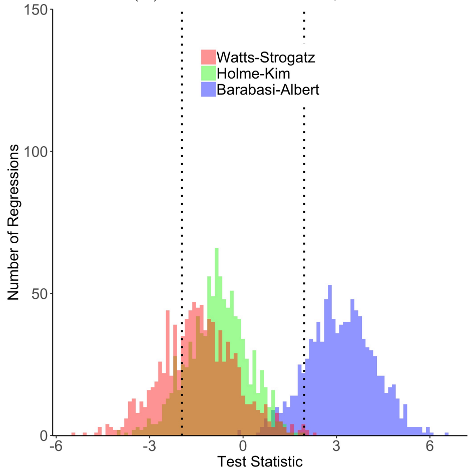

(c) Network Control

Note: Results for other variables from Equation 1 and threshold variables. 1,000 samples, all values of r. (a) Barabási-Albert and Holme-Kim networks display consistent results for network density. Watts-Strogatz produces contradictory results. (b) Regardless of network type, no consistent effect for global clustering exists.

S4.1 The Effect of Clustering on Repression

Figure A9 shows the results when selecting only Barabási-Albert networks where $\alpha = 3$, making it equivalent to a Holme-Kim network with less clustering. While the main results hold, a new result also emerges. Repression on Barabási-Albert networks does not succeed at low values. On these networks, it has no effect until approximately 7.5% of protesters are removed. On Watts-Strogatz, it almost always succeeds; on Holme-Kim networks, it succeeds about half the time. This delay is not apparent in the manuscript's figures because they sample all values of α. Local clustering (the Holme-Kim model) makes repression more successful.

Fig A9. Results Hold When Holme-Kim, Barabási-Albert Networks are Most Similar

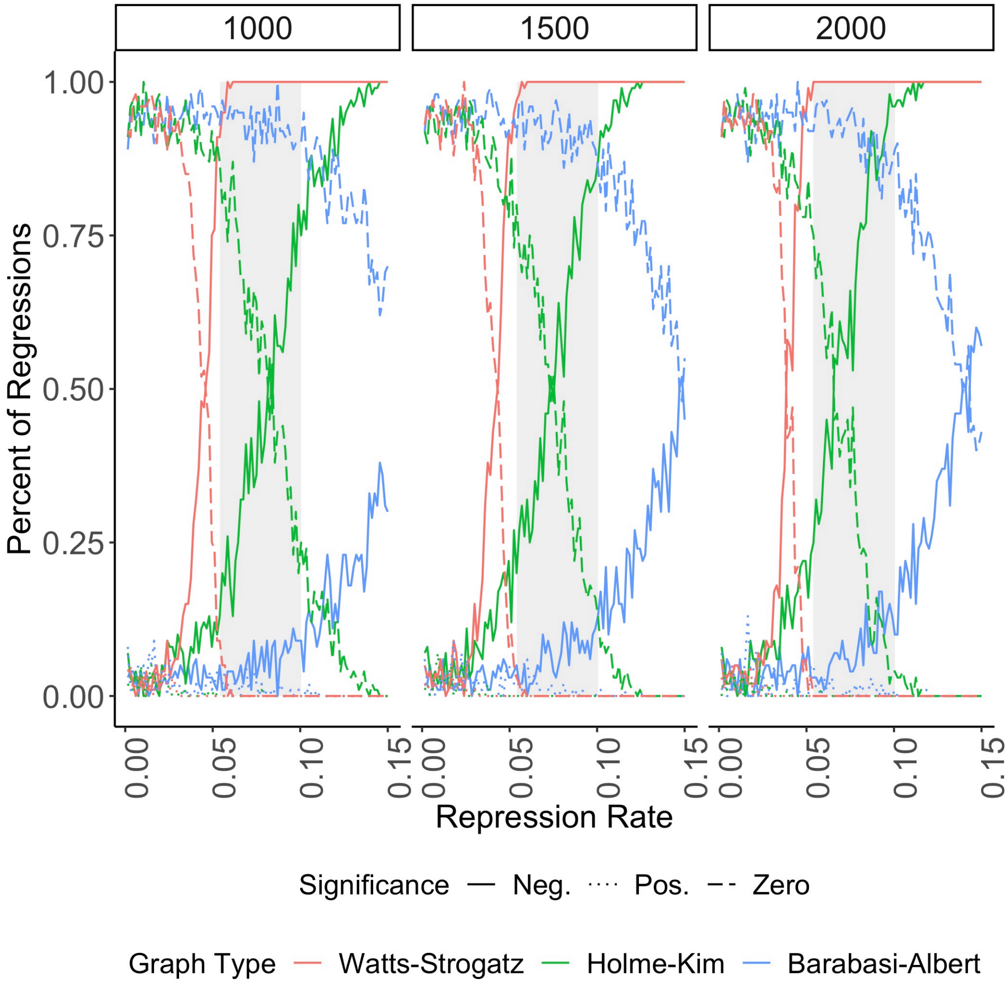

Note: The y-axis is the percent of regressions which find no relationship or a statistically significant, positive or negative, one. The facet label is the size of the sample.

Figure A10 shows the heterogenous effects from varying the *network structure control* variable. Because $2 \leq \alpha \leq 3$ while $0 \leq p, P_t \leq 1$, the x-axis is $\alpha - 2$ for the Barabási-Albert results. As $\alpha \to 3$, network degree centrality becomes less skewed. As $p \to 1$, the Watts-Strogatz model progresses from a wide ring to a small-world to a completely random network. In the Holme-Kim model, clustering increases as $P_t \to 1$. The y-axis for these results is the percent of time the network structure control variable is statistically significantly different from 0 (positive or negative) or not.

Fig A10. Varying α (Barabási-Albert), p (Watts-Strogatz), and P_t (Holme-Kim)

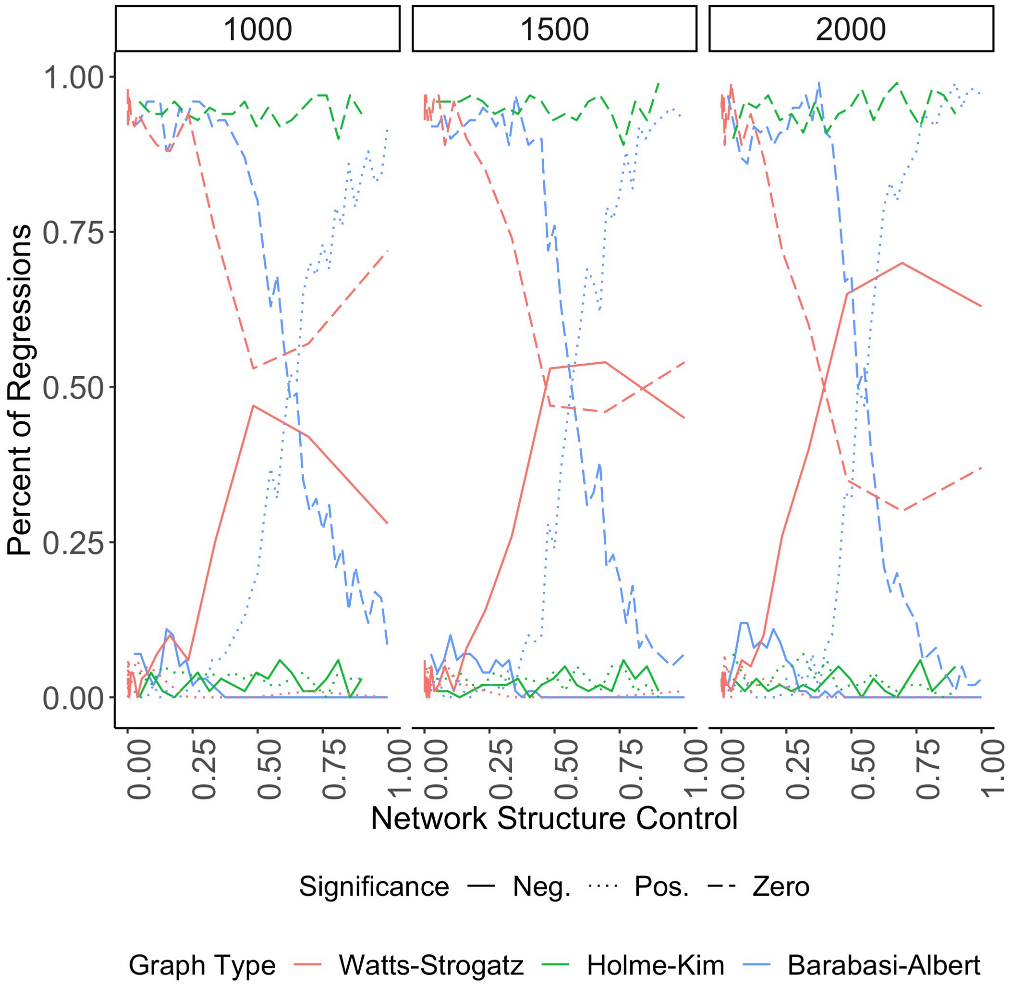

Note: The y-axis is the percent of regressions which find a statistically significant (positive or negative) or zero correlation between the network structure control and protest size. $p, P_t = x,\ \alpha = x + 2$. The facet label is the size of the sample.

Figures A10 reveals several interesting dynamics. Watts-Strogatz networks do not become more likely to have protests diffuse as they transition from regular to small-world ($p \leq .1$), but as local clustering approaches that of a random network ($p \rightarrow 1$), the probability of protest succeeding increases then decreases. Protests on Holme-Kim networks are not sensitive to increases in local clustering. The scaling parameter of Barabási-Albert networks exhibits a similar puzzle as the repression rate: as it increases (as network centrality becomes less skewed), protest becomes more likely to succeed. When the network structure control parameter equals 0 for the Holme-Kim and the Barabási-Albert, they are the same model and thus produce the same inferences. The lack of clustering as the Barabási-Albert's degree becomes less skewed suggests the inhibiting effect local clustering has on the spread of protests.

Appendix References

1. Rasler K. Concessions, Repression, and Political Protest in the Iranian Revolution. American Sociological Review. 1996;61(1):132–152.
2. Moore WH. The Repression of Dissent: A Substitution Model of Government Coercion. Journal of Conflict Resolution. 2000;44(1):107–127. doi:10.1177/0022002700044001006.
3. Osa M, Corduneanu-Huci C. Running Uphill: Political Opportunity in Non-democracies. Comparative Sociology. 2003;2(4):605–629.
4. Muller EN. Income Inequality, Regime Repressiveness, and Political Violence. American Sociological Review. 1985;50(1):47–61.
5. Schock K. People Power and Political Opportunities: Social Movement Mobilization and Outcomes in the Philippines and Burma. Social Problems. 1999;46(3):355–375. doi:10.1525/sp.1999.46.3.03x0250p.
6. Carey SC. The Dynamic Relationship Between Protest and Repression. Political Research Quarterly. 2006;59(1):1–11. doi:10.1177/106591290605900101.
7. Francisco RA. The Relationship between Coercion and Protest: An Empirical Evaluation in Three Coercive States. Journal of Conflict Resolution. 1995;39(2):263–282. doi:10.1177/0022002795039002003.
8. Francisco RA. After the Massacre: Mobilization in the Wake of Harsh Repression. Mobilization: An International Journal. 2004;9(2):107–126.
9. Gurr TR, Moore WH. Ethnopolitical Rebellion: A Cross-Sectional Analysis of the 1980s with Risk Assessments for the 1990s. American Journal of Political Science. 1997;41(4):1079–1103.
10. Francisco RA. Coercion and Protest: An Empirical Test in Two Democratic States. American Journal of Political Science. 1996;40(4):1179–1204.
11. Snyder D, Tilly C. Hardship and Collective Violence in France, 1830 to 1960. American Sociological Review. 1972;37(5):520–532.
12. Ritter EH, Conrad CR. Preventing and Responding to Dissent: The Observational Challenges of Explaining Strategic Repression. American Political Science Review. 2016;110(1):85–99. doi:10.1017/S0003055415000623.
13. Ritter EH. Policy Disputes, Political Survival, and the Onset and Severity of State Repression. Journal of Conflict Resolution. 2014;58(1):143–168. doi:10.1177/0022002712468724.
14. King G, Lowe W. An Automated Information Extraction Tool for International Conflict Data with Performance as Good as Human Coders: A Rare Events Evaluation Design. International Organization. 2003;57(03). doi:10.1017/S0020818303573064.
15. McAdam D, McCarthy J, Olzak S, Soule S. Dynamics of Collective Action Dataset; 2009. Available from: https://web.stanford.edu/group/collectiveaction/cgi-bin/drupal/.
16. Salehyan I, Hendrix C, Hammer J, Case C, Linebarger C, Stull E, et al. Social Conflict in Africa: A New Database. International Interactions. 2012;38(4):503–511.

17. Hill Jr DW, Jones ZM. An Empirical Evaluation of Explanations for State Repression. American Political Science Review. 2014;108(03):661–687. doi:10.1017/S0003055414000306.

18. Steinert-Threlkeld ZC. Spontaneous Collective Action: Peripheral Mobilization During the Arab Spring. American Political Science Review. 2017;111(02):379–403.

19. Tullock G. The Paradox of Revolution. Public Choice. 1971;11:89–99.

20. Granovetter M. Threshold Models of Collective Behavior. American Journal of Sociology. 1978;83(6):1420–1443.

21. Epstein JM. Modeling civil violence: An agent-based computational approach. Proceedings of the National Academy of Sciences. 2002;99(3):7423–7250.

22. Lohmann S. The Dynamics of Informational Cascades: The Monday Demonstrations in Leipzig, East Germany, 1989-91. World Politics. 1994;47(1):42–101.

23. Pearlman W. Emotions and the Microfoundations of the Arab Uprisings. Perspectives on Politics. 2013;11(02):387–409. doi:10.1017/S1537592713001072.

24. Centola D, Macy M. Complex Contagion and the Weakness of Long Ties. American Journal of Sociology. 2007;113(3):702–734.

25. Rutherford A, Cebrian M, Dsouza S, Moro E, Pentland A, Rahwan I. Limits of social mobilization. Proceedings of the National Academy of Sciences of the United States of America. 2013;110(16):6281–6. doi:10.1073/pnas.1216338110.

26. McAdam D. Recruitment to High-Risk Activism: The Case of Freedom Summer. American Journal of Sociology. 1986;92(1):64–90.

27. Opp KD, Gern C. Dissident Groups, Personal Networks, and Spontaneous Cooperation: The East German Revolution of 1989. American Sociological Review. 1993;58(5):659–680.

28. Tufekci Z, Wilson C. Social Media and the Decision to Participate in Political Protest: Observations From Tahrir Square. Journal of Communication. 2012;62(2):363–379. doi:10.1111/j.1460-2466.2012.01629.x.

29. Onnela JP, Saramäki J, Hyvönen J, Szabó G, De Menezes MA, Kaski K, et al. Analysis of a large-scale weighted network of one-to-one human communication. New Journal of Physics. 2007;9(6). doi:10.1088/1367-2630/9/6/179.

30. Watts DJ, Strogatz SH. Collective dynamics of 'small-world' networks. Nature. 1998;393(6684):440–2. doi:10.1038/30918.

31. Barabási AL, Albert R. Emergence of Scaling in Random Networks. Science. 1999;286(October):509–513.

32. Yule G. A Mathematical Theory of Evolution Based on the Conclusions of Dr. J.C. Willis. Philosophical Transactions of the Royal Society of London (Series B). 1925;213:21–87.

33. Holme P, Kim BJ. Growing scale-free networks with tunable clustering. Physical Review E - Statistical Physics, Plasmas, Fluids, and Related Interdisciplinary Topics. 2002;65(2). doi:10.1103/PhysRevE.65.026107.

34. Kwak H, Lee C, Park H, Moon S. What is Twitter, a Social Network or a News Media? In: International World Wide Conference. Raleigh: ACM Press; 2010. p. 591–600.

35. Newman MEJ. Power laws, Pareto distributions and Zipf's law. Contemporary Physics. 2005;46(5):323–351. doi:10.1080/00107510500052444.

36. Mandelbrot B. Fractals and Scaling in Finance: Discontinuity, Concentration, Risk. New York City: Springer; 1997.
