# Supplementary material for: How social networks affect the repression-dissent puzzle
Source: PLoS One. 2021 May 6;16(5):e0250784. doi: 10.1371/journal.pone.0250784 (PMC8101725; doi:10.1371/journal.pone.0250784)
Supplement: Attachment — Submitted filename: PLOS_One_RevisionLetter.docx [file pone.0250784.s002.docx]

Dear Editor,

Thank you for finding two reviewers who gave excellent feedback and providing us the opportunity to revise the paper to address the reviewers’ concerns. **Their comments are in bold**, and our responses follow each comment.

**REVIEWER 1**

**First of all, it is somewhat hard to follow the main line of thought when reading the paper. I am sure that future readers would reward some restructuring. The current structure is Introduction -> Results (Repression-Dissent Puzzle, The Model, Repression) -> Discussion. The text under "The Model" (that includes the majority of the text) might be separated with more sub-heading to help following the different applications of the model.**

- The reviewer makes an excellent point, as no academic paper has just three sections. Realizing that we crammed too much into the previous Results section, we have now split it into several. After the Introduction, we now have “The Persistence of the Repression-Dissent Puzzle”, which was previously the first subsection of Results. We then create another section, “Our Model”, that explains the paper’s simulation setup. The following section, “Simulation Results”, is what “Regression Results” used to be; we have also added an overview paragraph to the start of this section to enhance clarity. Subsections of “Simulation Results” now explain the regression model (including justification of the parameters, a point to which we return later in response to R2), the primary results, results as a function of the repression rate and sample size, more realistic network parameters and repression rates, alternative repression strategies, and robustness checks. We have then made previous S5 its own section in the manuscript, and we conclude with the discussion. Overall, the paper now has six parent sections versus three in the original version.

**Also, I found the information in S5 (Implications for Understanding Repression and Protest) very important. Including more direct references to the arguments in S5 in the main text could be useful.**

- We thank the reviewer for this encouragement! Previous S5 is one of our favorite sections, but we found some audiences did not like it in the manuscript. We have now made it the penultimate section, after the Results and before discussion. While bringing S5 into the manuscript, we undertook some editing for clarity. S5.1 and 5.2 are now the subsection “Repression and Loyalty”.

**A short explanation could added to the text describing the models where the network configuration parameters were adjusted to real social networks. Here my concern is how much the authors treat evidence from Twitter and email networks as proxies for real social networks? How were p and mt values adjusted to the clustering of the two networks?**

- To clarify the previous writing, we have split the paragraph that explains these values, the one starting “Figure 5 replicates Figures 3 and 4 but with network […]”, into two. The end of the new first paragraph clarifies that we are only looking at specific subsets of each of the three network types: we did not adjust any values, but rather used ones that had already been set in other iterations of the simulations. The second paragraph then starts with the same justification as from the original paper. To ground our parameter choices in non-electronic data, we then emphasize that Christakis and Fowler (2007) did not use social media data and cite two studies that used cell phone data to measure social networks: Stopczynski et al. 2014 and Schalpfer et al. 2014.

**Some adjustments in the size of the texts and some other minor changes in the figures should be made. (E.g. X axis text in Figure A3 and A4, more horizontal space between Fig6ab and Fig6cd; Fig5a and Fig5b ) In several figures the line type for insignificant models is literally indistinguishable from the positive ones.**

- We appreciate that Reviewer 1 asked us to improve the legibility of our figures. On all figures, not just A3 and A4, we have made the x-axis text vertically oriented so that one panel’s “0.15” does not overlap with the next one’s “0.00”. For all figures, we also have changed the linetype for positive results from dash-dash-dot to dotted, so it is now more clearly distinguishable from the zero linetype, which is still a long dash.
- Regarding horizontal space for Figures 5 and 6, we could not discern if the reviewer wants the subfigures in each row more separated from each other (a more space from b, c from d) or the top row (a, ab) to be more separate from the bottom (cd). Since the latter interpretation better fits both figures, we interpreted the request as the latter. We there added vertical spacing between the top and bottom rows of Figures 5 and 7; this paper’s Figure 7 is the original paper’s Figure 6.
- If we misinterpreted this comment from R1, we are happy to update the figure spacing a different way.

**Finally, I am not aware how this should be handled, but submission rules for Plos One state that "A Data Availability Statement describing where the data can be found is required at submission." Currently there is no reference in the text to the location of the data.**

- We thank the reviewer for noticing this simple oversight. We have now added a Data Availability statement after the discussion section and before Acknowledgments. The simulation data, MMAD, MM, and NAVCO 3.0 are now available with a DOI number.

**REVIEWER 2**

Overall Reviewer 2’s comments focus on making the paper’s argument clearer and emphasizing the importance of networks throughout. These insightful comments are much appreciated, and we believe the paper is now much stronger.

**But the paper is very hard to follow. For example, in the second paragraph on page two, in one paragraph, the authors say (1) the repression dissent puzzle might be driven by sampling issues; (2) so the authors will model protests on networks; (3) contextual factors like network structure and repression severity determine the success of protests, but inference is still sensitive to episodes. These are three very different sentences wedged into one paragraph. If the problem is about sampling, then is the solution about research design? Why networks?**

- We thank R1 for noticing the unclear writing. To introduce networks better, we now start the second sentence of paragraph 2 with, “Because social networks are a key determinant in individuals’ decision to protest,”. We have also removed the last sentence of paragraph 2, “While contextual factors[…]”, and replaced it with a simpler statement about the repression-dissent puzzle always emerging.

**The language here is so difficult to follow. By the end of page 2 the authors explain how their regressions will work, but it still isn't clear (a) why networks? when most of the lit has not studied networks-- this is a big leap to sell to your audience (repression/nonviolence scholars). Why is THIS decision important? And (b), what does it mean that the 'repression-dissent puzzle' here is 'fundamental' to the 'empirical approach' used in studies of protests and repression? The prose is hard to parse. Be clear, be straightforward, then we can interpret the meaning of your statistical analysis more clearly.**

- We thank the reviewer for forcing us to motivate the use of networks more. We have therefore added a new third paragraph in the introduction. It explains why we model protests with networks. It shows that a lot of the literature has used networks, so we are not out of the mainstream.
- We had called the puzzle “fundamental” just to emphasize how persistent it is. But we have now removed that sentence, the end of the third paragraph of the original paper.
- We have added this emphasis sentence at the end of the first paragraph of the Analysis Method section: “This modeling is suggestive of the type of network features future data collection should try to record and some researchers have started to use.”
- We have also added a new concluding paragraph to the introduction that situates this paper in the growing literature that is using networks to understand protest and intrastate conflict. With these changes, we hope R2 agrees that the new introduction has more strongly motivated the use of networks.

**I liked the author's summary in Table 1, and discussion of the relevant literature but, again, there is very little attempt to explain to readers why the authors are going to take a 'network' approach. Though I did not re-read the studies cited, it seems they are predominantly event or country level data.**

- We hope that the new paragraph three addressed this astute concern.
- We have added a note to Table 1 emphasizing that none of the studies use network measures. We have also added a two-sentence paragraph at the end of the “The Persistence of the Repression-Dissent Puzzle” section that emphasizes the lack of network data in previous studies.

**The discussion of the model should be moved to its own section. More discussion and depth is required as to why these three networks are chosen for the study.**

- This request is much appreciated and in line with Reviewer 1’s. We now have “Our Model” as its own section.
- The first paragraph of Our Model now more clearly explains why we chose the three models. Human social networks have three features: local clustering, short average path lengths, and highly skewed degree distributions. No model incorporates all 3, so we choose 3 that incorporate some combination of these features.
- We have elected to keep much of the justification in Section 2.3 because we brought Section S5 into the manuscript in response to R1. We are more than happy to bring S2.3 into the manuscript as well if that is desired.

**I know the authors moved some of this reasoning to the supplementary materials, but they could require these first 1-8 pages to be much clearer leading up to this point, especially since this seems to be essential to the main takeaway of the paper. Leading with networks, and their structure, early on will give the reader a better intuition about the paper.**

- We again thank R2 for pushing us to make the paper more coherent. With two new paragraphs in the introduction about social networks, the final two sentences of “The Persistence of the Repression-Dissent Puzzle”, and justifying the three models better at the start of “Our Model”, we believe the paper is now much more clearly about networks and so gives the reader a better intuition about the paper.

**The modeling section is clearer than other parts of the paper, but the empirical section can be revised to share more information about the data choices the authors made. There needs to be a clearer justification of the network parameters used in the model (eq 1) and the sensitivity of the results to these covariates.**

- R2 is again correct that the paper is opaque. The section to which R2 refers is now the Analysis Method section, and we have added a new paragraph that explains why we chose the variables in Equation 1. This paragraph, starting “These variables were chosen to capture” explains that each is included for theoretically motivated reasons, and citations are provided to relevant work.
- We apologize to R2 for not making the “sensitivity to network parameters” clearer. In the original paper, Footnote 7 mentioned a regression with two other variables and pointed the reader to their corresponding figures in the SI. That sentence is now brought into the paragraph starting “We have also confirmed that the results” on page 16. That paragraph also mentions three more parsimonious models we ran but whose results we did not include because nothing changed. The following three figures show the results for 3 models: (1) only repression as an IV; (2) (1) + initial size; and (3) model (2) + density, mean degree, and neighborhood clustering. Those figures are now shown in order below.
  1.

  2.

  3.

- We prefer to leave those figures out of the SI but are happy to add them if R2 prefers.

**Further, it is unclear here which data the authors chose and why. Did they work with all the data listed in Table 1? Only a select amount? Again, the choices are buried.**

- We thank R2 again for asking us to clarify data sources. At no point for model or results do we use the data in Table 1. We have added a sentence to the end of the first full paragraph on page 6 (“For each regression,”) that says, “At no point do we use any data from the studies in Table 1: except to determine realistic repression rates, the rest of the paper only analyzes the simulation data.”

**It is unclear what the contribution is, following the simulations. If the problem of studying the repression-dissent puzzle is about measurement, then can the authors spell out exactly their contribution to solving this? On page 24 the authors suggest, the solution to the problem is to embrace a new research design, one that collect data on networks rather than events. This seems reasonable, and if it is the main argument then I encourage the authors to re-write it as the through-line of the paper. The authors can then use a burgeoning literature on intrastate conflict networks to support / compliment this idea (Larson, Dorff, Gade, Minhas, Cunningham etc).**

- R2 is correct that the original paper did not make the contribution clear, and we have taken several steps to rectify this hole. First, the new last paragraph of the introduction explicitly calls on researchers to reconsider their research design by including more network data. We show that some already are, and we provide citations (#s 31, 32, and 33) to the excellent work to which R2 pointed us. Second, bringing S5 into the paper as its own section (“Implications for Understanding Repression and Protest”) also emphasizes how to think about existing phenomena in network terms, a reorientation that we hope spurs new types of research.

Sincerely,

Shane Steinert-Threlkeld and Zachary C. Steinert-Threlkeld
